# Evidence to practice – lessons learnt in developing an implementation strategy for an online digital health intervention (Eczema Care Online)

**DOI:** 10.1186/s12913-024-12179-2

**Published:** 2025-01-31

**Authors:** Laura Howells, Kim S. Thomas, Miriam Santer, Ingrid Muller, Kate Greenwell, Amanda Roberts, Hywel C. Williams, Jane Harvey, Stephanie J. Lax, Natasha K. Rogers, Tracey H. Sach, Sandra Lawton, Mary Steele, Katy Sivyer, Julie Hooper, Amina Ahmed, Sylvia Wilczynska, Sinead Langan, Paul Leighton

**Affiliations:** 1 Centre of Evidence Based Dermatology, School of Medicine, University of Nottingham, Applied Health Services Research Building (Building Number 42), University Park, Nottingham, UK; 2 Primary Care Research Centre, Faculty of Medicine, University of Southampton, Aldermoor Health Centre, Southampton, UK; 3 School of Psychology, University of Southampton, Highfield, Southampton, UK; 4 The Rotherham NHS Foundation Trust, Rotherham, UK; 5 Public Contributor, Nottingham, UK; 6 Department of Non-Communicable Disease Epidemiology, London School of Hygiene and Tropical Medicine, London, UK

**Keywords:** Digital health intervention, Implementation, Atopic eczema, Normalisation process theory

## Abstract

**Background:**

Eczema Care Online (www.EczemaCareOnline.org.uk/) is an online self-management toolkit which includes tailored content for young people (13–25 years) and for parents of children that have eczema (0–12 years). Testing in two randomised controlled trials has shown that it is easy to use, cost effective and offers a sustained improvement in eczema symptoms.

Implementing Eczema Care Online outside of a funded research study and ensuring that it reaches those that will most benefit from is now a key challenge. This paper describes the lessons learnt from developing and delivering an implementation strategy.

**Methods:**

Data from systematic reviews, stakeholder consultation meetings, interviews with trial participants, intervention usage data during the trial, and existing eczema information websites informed our implementation plan. Using Normalisation Process Theory, an implementation plan combined these findings with practical, context-specific actions to encourage wider adoption of the intervention.

**Results:**

Data was successfully mapped to the four constructs of Normalisation Process Theory, and factors and processes that encourage implementation identified. These include: promoting how Eczema Care Online is different to other sources of information; aligning to and embedding in existing eczema resources (from charities and healthcare providers); simplifying aspects to aid ease of use; and, highlighting evidence that shows that Eczema Care Online works.

Key lessons in developing an implementation strategy include 1) start implementation work early 2) maintain flexibility to explore multiple routes to implementation 3) use secondary data sources 4) balance theory with practicalities 5) consider longer-term maintenance beyond the life of the research project.

**Conclusion:**

Implementation planning is a key stage of the research process that is often not adequately resourced. Implementation planning ensures effective interventions developed and evaluated in research studies are utilised in everyday practice.

## Introduction

### The implementation challenge

Research has the potential to improve healthcare, but the reality is that much health research is not taken up in healthcare settings or, when it is, it may take a long time to have an impact [1, 2]. This is often referred to as the ‘implementation gap’—things that are shown to work in research studies often work less well or not at all in real-world contexts [3, 4]. This has been identified as a sources of avoidable research waste [5–7]; it is estimated that as much as 85% of medical research makes no difference to end users [8].

To improve the chances of an innovation having a positive impact research communities are increasingly focusing upon implementation, demonstrating a concern for how implementation happens or can be supported [9]. Variously described as ‘implementation science’ or ‘knowledge mobilisation’ this is an explicit focus upon “the process of moving knowledge to where it can be most useful” [9], this often compliments research focused upon clinical efficacy and/or effectiveness.

Normalisation Process Theory (NPT) is one way of understanding the challenges of implementation [10, 11]. NPT focuses upon the ‘work’ that individuals do to ‘routinely embed’ complex interventions in organisational and/or professional contexts. It considers those factors which support (or inhibit) new ways of working becoming ‘normal’ and routine [11]. The theory outlines four key processes, which are included in Table 1.

**Table 1:** Normalisation Process Theory (NPT) four key processes [11]

1) Coherence; how people make sense, both individually and collectively, of the work to be done
2) Cognitive participation; how people engage with the work
3) Collective action; how people enact the work
4) Reflexive monitoring; how people appraise the work to be done

Despite a growing recognition that implementation should be considered at all stages of intervention development and evaluation [12–14] efforts are not regularly documented or reflected upon. Limited examples demonstrate the importance of planning implementation from the outset, building explicit strategies for implementation, and building implementation upon appropriate theory [15–17]. Here we share lessons learnt in developing an implementation plan for Eczema Care Online, these lessons will have relevance for others developing digital health interventions.

### Eczema care online

Eczema Care Online (www.EczemaCareOnline.org.uk) is a self-management toolkit which provides tailored support to (i) parents/carers of children with eczema (0–12 years) and (ii) young people who are starting to manage their eczema (13–25 years) [18, 19]. It was developed to address the challenge that individuals face when seeking information about eczema, and the variable quality of information that is available online [20, 21]. It was developed using current evidence and the person-based approach [18, 19].

**Fig. 1:**
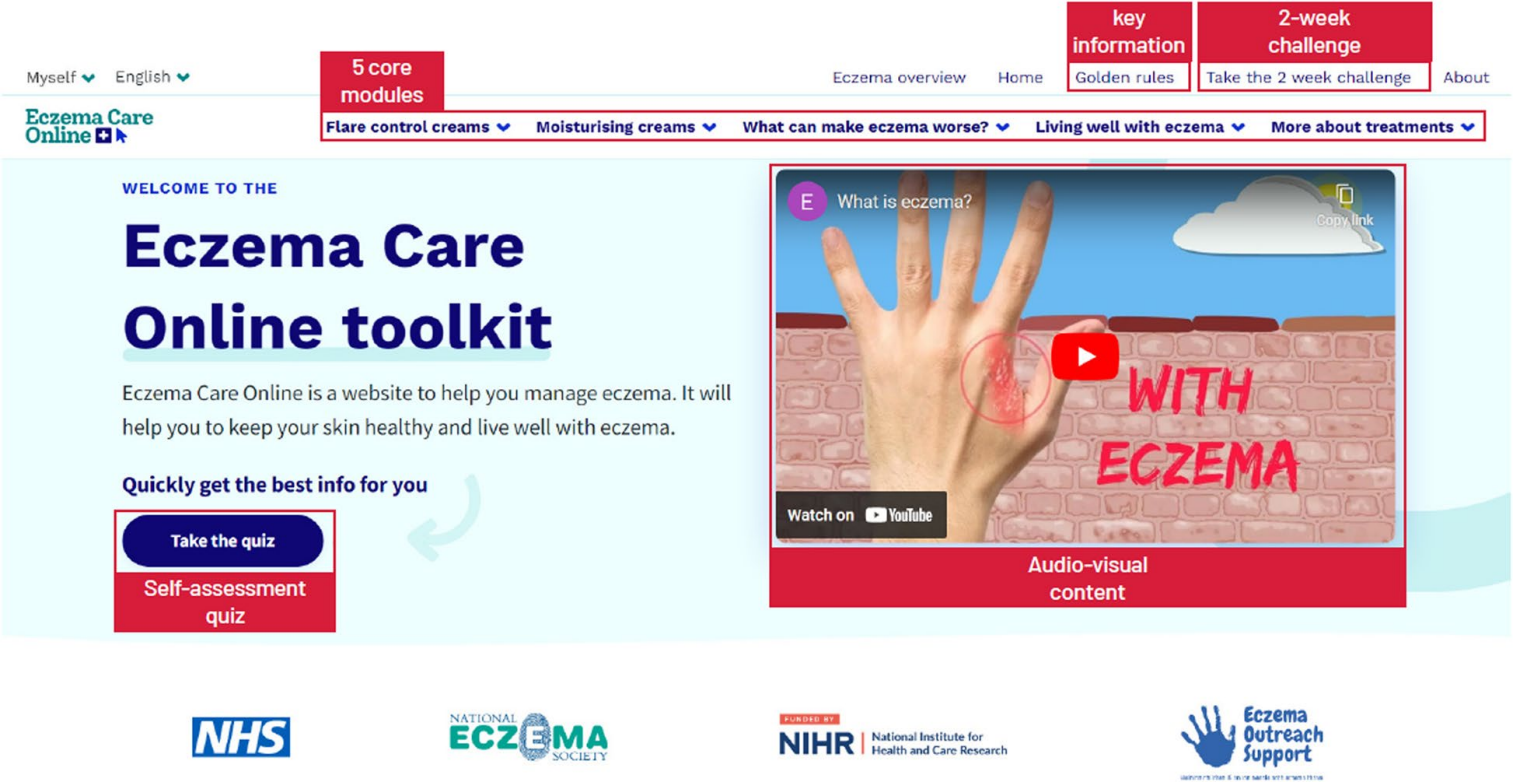
Annotated screenshot of Eczema Care Online

Eczema Care Online includes interactive audio-visual content which presents key information about eczema as well as behaviour change strategies. It includes a brief eczema assessment, video stories, and advice from others that have experienced eczema. Figure 1 highlights some key features.

Randomised controlled trials recruiting from primary care in the UK demonstrated that access to Eczema Care Online led to a small but sustained improvement in eczema symptoms compared to usual care [22]. Nested qualitative interviews suggest that users found the interventions easy to use, relatable, and trustworthy, and perceived that the intervention helped them to better manage their eczema [23]. A quantitative process evaluation concluded that engagement was high, and that the intervention required minimal time commitment [24]. Health economic analysis suggests cost-effectiveness [25]. Together, this evidence suggests that adoption of this intervention in healthcare or community settings could have benefits for people with eczema.

Self-management digital interventions such as Eczema Care Online bring with them distinct implementation challenges – how do they sit with existing systems/knowledge, do they disrupt relationships with healthcare professionals, how is the technology received/managed [15]? Successful implementation of Eczema Care Online is challenging as it requires adoption at patient/family, clinical, and organisational levels. Individuals with eczema, or parents/carers of children with eczema, need to be able to use the intervention as well as healthcare professionals who are required to advocate and promote its use.

### Aim

To describe the lessons learnt from developing and delivering an implementation strategy for an online digital health intervention for eczema self-management.

### Objectives

1. To illustrate the value of using existing data to support implementation.
2. To illustrate the importance of using a theoretical framework to support implementation.
3. To describe the lessons learnt for other research teams wanting to invest in implementation within their programme of research.

## Methods

Guided by the work of Ross et al. [15] a range of information sources were drawn upon to inform an implementation strategy for Eczema Care Online. This took place alongside the development [18–21] and testing [22–25] of Eczema Care Online, with data and information generated in these processes informing implementation planning. Information was structured and interpreted using NPT as a theoretical framework for implementation.

### Data used to inform implementation

#### Data source 1: Review of published literature

Alongside developing and testing Eczema Care Online a Cochrane systematic literature review was conducted on strategies for using topical corticosteroids [26]. This was to ensure that Eczema Care Online content was up to date; review findings were compared to existing UK guidelines to identify new, important insight [27–29].

#### Data source 2: Stakeholder consultation

Stakeholder consultation took place throughout the 5-year research programme which encapsulates the development [18–21] and testing [22–25] of Eczema Care Online. At different timepoints stakeholders were engaged to support the interpretation and implementation of the research findings. Stakeholders included: researchers and clinicians involved in managing and delivering Eczema Care Online; clinicians recruited via professional networks (such as the Society of Academic Primary Care Dermatology Special Interest Group); existing public and patient research groups (such as the Centre of Evidence Based Dermatology Patient Panel); and members of the public recruited via other patient-interest groups (such as the National Eczema Society and Eczema Outreach Support). Some stakeholders (especially those involved in the delivery and management of Eczema Care Online) attended on multiple occasions. Implementation was considered at the following meetings:

1. A 1-day face-to-face meeting with 34 attendees including researchers (*n* = 11), healthcare professionals (*n* = 13), patient partners/organisations (*n* = 10) (Sept 2019). This meeting involved reviewing preliminary Cochrane systematic review findings.
2. Five 90-min online meetings targeted to specific stakeholder groups to refine key messages about eczema treatments. These were recruited via the mechanisms and networks listed above with no limit to the number of participants possible at each meeting. Thirty stakeholders took part in these meetings: primary care (*n* = 4), secondary care (*n* = 7), pharmacy (*n* = 5), people with eczema/patient organisations (*n* = 7), and parents of children with eczema/patient organisations (*n* = 7) (March 2021).
3. An additional 2-h online meeting with 18 individuals or patient organisation representatives gathered views from people concerned about a safety concern of eczema treatments termed ‘topical corticosteroid withdrawal’ (May 2021).
4. A 1-day results reveal meeting (16 in person and 16 online attendees) including researchers, healthcare professionals, patients, and patient organisations (April 2022) where implementation actions were a key discussion point.

#### Data source 3: Interview data

Interviews generated alongside the randomised controlled trials [23] were reanalysed with a more explicit concern for implementation. Forty interviews with trial participants (20 young people and 20 parents) were deductively coded to an analytic framework [30] based upon the four core NPT constructs. Coding was led by LH, supported by PL, and shared regularly with the wider team.

#### Data source 4: Usage statistics

During the testing of Eczema Care Online the online platform automatically recorded usage: frequency of visits, duration of visits, hits on specific bits of the platform, etc. This usage data was collected from the intervention arm of each trial and included 171 parents and carers and 168 young people who were given access to Eczema Care Online. A review of this data identified the most frequently used aspects of the intervention which allows a refining of Eczema Care Online prior to implementation. For example, aspects rarely used might be edited or removed, more frequently used aspects might be positioned to make them easier to identify and access.

#### Data source 5: Existing eczema websites

To understand how Eczema Care Online fits within the landscape of currently available online information for eczema, we identified what other online eczema resources are available. We looked for: a) websites or webapps providing information about the cause, management, treatment or living with eczema; b) both public facing and targeted to professionals; c) freely accessible to members of the public. We did not consider any resources that required registration or payment to access.

Beyond those resources that the authors were already aware of (such as the NHS or national organisation websites) others were identified by searching www.google.co.uk (3rd-9th Feb 2022). A variety of simple queries relating to eczema management were searched (to replicate how an average google user may find resources), a country filter for the United Kingdom was used to limit hits. This search was not intended to be comprehensive or replicable.

Two researchers (LH, PL or NR) independently reviewed website content and provided a subjective rating of the ‘unique selling points’ of each.

### Theoretical framework

**Fig. 2:**
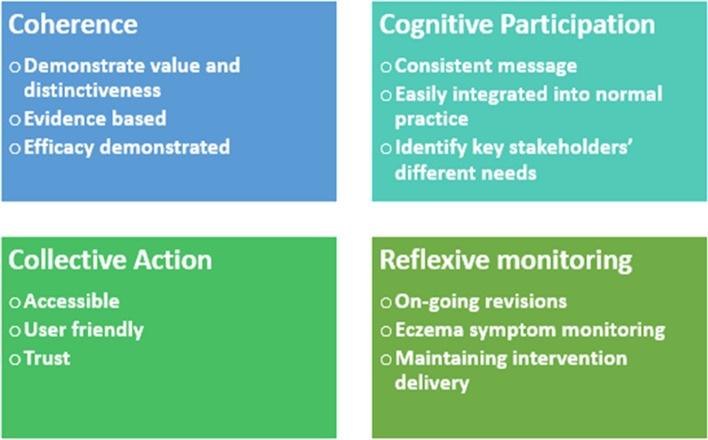
Framework to support implementation planning

We created a theoretical framework that articulates a context specific interpretation of NPT. This includes the four key NPT constructs with examples of processes considered to be supportive of the successful implementation of Eczema Care Online (Fig. 2).

Populating this framework using data generated during the development and testing of Eczema Care Online informs a practical plan for implementation. Building this plan potentially requires a negotiation between NPT constructs and practical, contextual considerations. For example, NPT analysis might identify the National Health Service (NHS) as a ‘trusted source’ of information (NPT—collective action), but implementing Eczema Care Online via the NHS might require an assessment of which NHS sources are most relevant or practical.

## Results

**Fig. 3:**
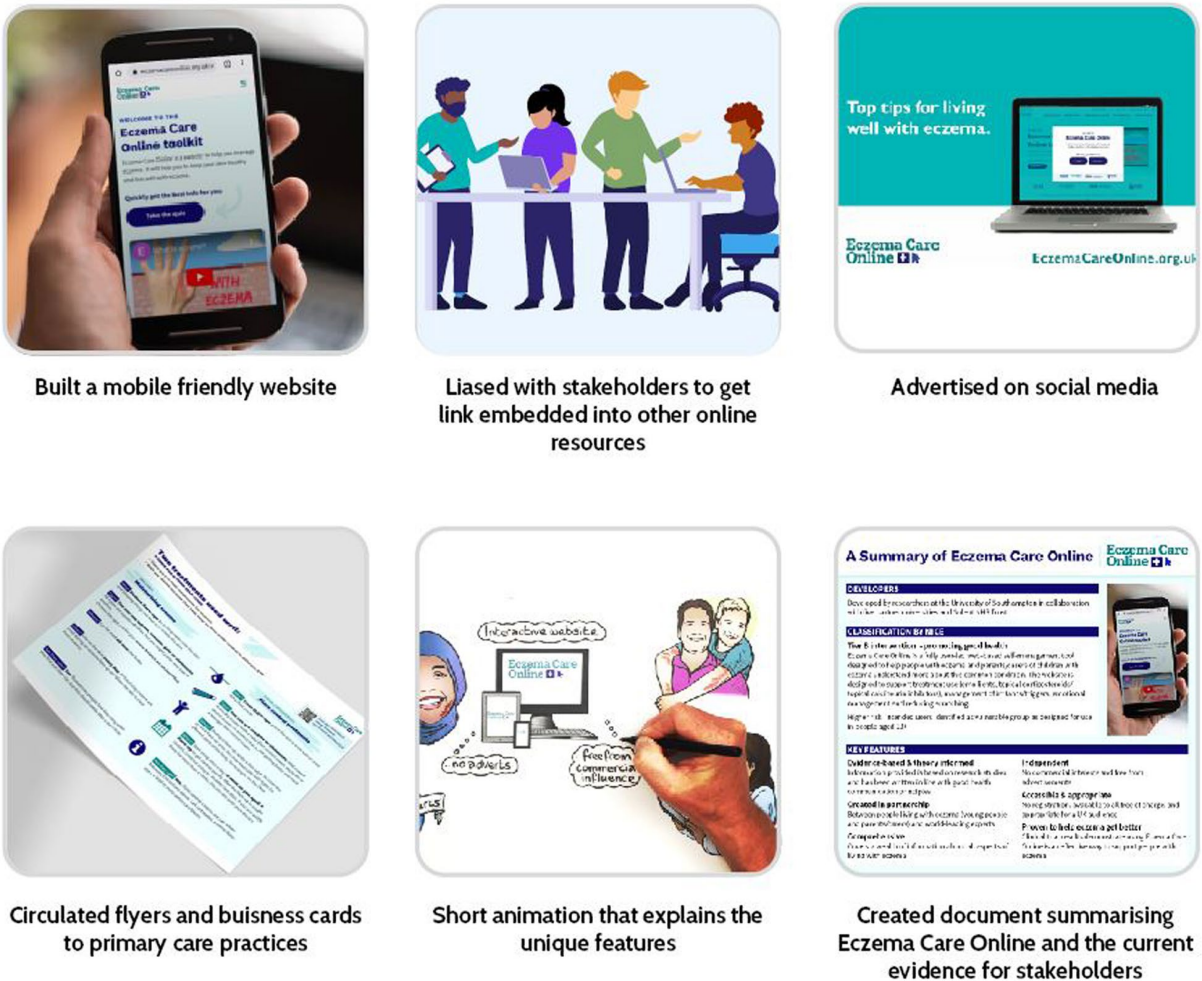
Key outputs supporting implementation

Through the development and testing of Eczema Care Online several avenues for implementation were identified and/or utilized (Fig. 3).

Organised around the four NPT constructs here we present how our understanding about implementation developed. For each NPT construct we present what we learned about the implementation challenge, and those consequent implementation actions and strategies which were developed and/or proposed.

### Coherence

#### Learning

Stakeholder meetings reinforced that Eczema Care Online is offering something that is distinct and potentially valuable to users and healthcare professionals. Process evaluation data highlighted that users felt that Eczema Care Online offers something different:

*“The ECO* [Eczema Care Online]* website is great, like I said, because it looks at the family as a whole and your life as a whole and the whole – management of it in your life […] This is very much part of your life, this is how we can help you, like the whole nursery stuff and the school stuff, like – that’s not on the NHS website” – Parent, aged 34, female, White British*

Our assessment of existing websites identified a crowded marketspace and a subsequent need to emphasise how Eczema Care Online is distinctive in content, quality and purpose.

#### Actions

**Fig. 4:**
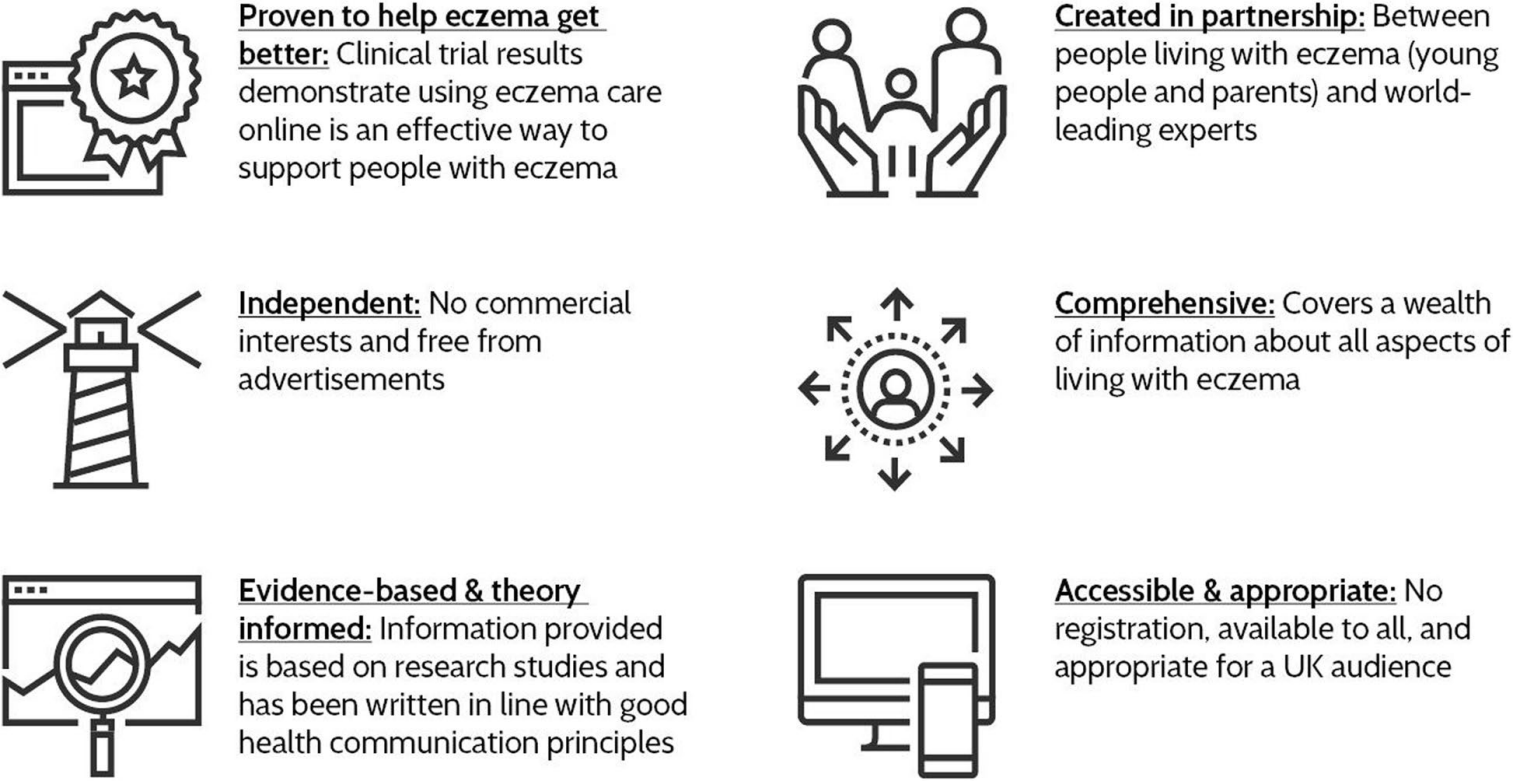
Value Propositions – selling points for the implementation of Eczema Care Online

Marketing literature proposes that ‘value propositions’ play a critical role in communicating how a company aims to provide value to its customers [31]. We constructed ‘value propositions’ using features that our analysis suggested were distinctive and valuable about Eczema Care Online (Fig. 4).

A ‘unique selling point’ of Eczema Care Online, compared to other eczema websites, was that it is ‘proven to help people with eczema’. Marketing materials emphasised this. For example, we created an animation (https://www.bmj.com/content/379/bmj- ID="EN1">2022–072007#media-1) and social media advertisements that promoted the proven efficacy of Eczema Care Online.

## Cognitive participation

### Learning

Users in the process evaluation were generally willing to engage with the platform and found it easier than other routes to access information about eczema (e.g., challenges accessing General Practice, overwhelmed by information via search engines).

However, there was a concern from some stakeholders (public as well as healthcare professionals) that Eczema Care online could ‘add to noise’ in an online space that is already saturated with ‘mixed messages’. Eczema Care Online needs to be up to date and should reinforce, rather than undermine, other trusted sources (e.g., guidelines, manufacturer packaging, healthcare professional advice to patients). The Cochrane review identified some key messages that are currently absent from guidelines, for example that once daily application of topical corticosteroids is adequate for most people.

Healthcare professionals stressed that they need simple and practical ways of incorporating messages into their practice, with electronic systems in primary care being increasingly important.

### Actions

We focused upon ensuring consistency and accessibility to promote the use of Eczema Care Online.

To support consistent and up to date messaging, we lobbied key places (e.g., UK guidelines) and disseminated Cochrane review findings widely to encourage shared messaging. We produced a ‘two treatments used well’ booklet and circulated this via primary care and some pharmacies and secondary care settings. We also contacted providers of electronic system providers so that links to Eczema Care Online could be accessed by primary care staff as well as sent in SMS to patients.

We contacted training providers so that Eczema Care Online could be embedded into healthcare training materials. We also identified secondary care ‘champions’ who are trusted sources of information for both secondary care and primary care colleagues. We developed template wording for healthcare professionals working in secondary care to add to ‘advice and guidance’ letters to primary care clinicians and made this available on our website.

## Collective action

### Learning

Process evaluation data demonstrated that ease of use is important to people, and that there were some aspects of the Eczema Care Online that needed improving to create an easier experience. For example, some people found the layout of the website confusing and struggled to find the content that they were interested in. Ease of access (i.e., no log in) was identified as being important to make Eczema Care Online more usable.

Stakeholder meetings with members of the public and with representatives of patient groups stressed that where information is found/recommended is important to its legitimacy. The value of signposting from healthcare professionals was recognised in the process evaluation:

*“they trust their GP* [general practitioner] *and they trust their doctors to give them advice that’s good for them. So having it through the NHS would be useful because people will trust the website, firstly, […] and will feel like, okay, well this is legit; they’re not giving them anything that could be wrong for them or could, you know, make their condition worse. […] people trust doctors and having it maybe, you know, people talking about it maybe on social media, so it makes it a bit more – a bit more – reachable.” (Young person aged 24, female, Indian)*

Some people were happy to act on information from less trusted but more convenient sources (e.g., social media), but in these case branding (such as ‘NHS recommended’) was important.

### Actions

In re-designing the website for implementation, we addressed users’ reported barriers to use. We ensured branding and logos of involved organisations were clear on the homepage and in advertising materials. We contacted numerous legitimate resources about embedding an Eczema Care Online link into their materials (e.g., NICE guidelines, professional body websites, eczema charity websites). To support engagement from trusted sources, we created a concise summary based on the NICE evidence summary framework to emphasise the legitimacy of the intervention [32]. We produced a public-facing booklet (described in Cognitive Participation) that could be distributed by healthcare professionals.

## Reflexive monitoring

### Learning

Healthcare professionals in the stakeholder meetings stressed that research data showing that Eczema Care Online produces a sustained benefit (i.e., trial findings) helps clinicians to feel confident that it works and supports their ongoing usage and recommendation of Eczema Care Online. They made a similar point about organisational support, that research evidence is essential for organisational buy in.

Users within the trial reported that completing questionnaires was helpful for monitoring changes to eczema and that they would value something similar in the future:

*“even just doing the questionnaires I found quite helpful because it makes me stop and assess how she’s doing and I don’t know that I would do that in quite the same way … in a different context. So, I think just kind of being prompted to think about it on a regular basis is quite helpful.”*

### Actions

Trial findings that Eczema Care Online improves eczema were included as a value proposition and emphasised in promotional material and active dissemination.

To allow users to engage in ongoing monitoring outside of a trial setting, we re-designed Eczema Care Online to include a link to My Eczema Tracker, an app that can be used to monitor eczema and improvements in symptoms (https://www.nottingham.ac.uk/research/groups/cebd/resources/my-eczema-tracker-app.aspx).

In future, further evidence will be needed to understand whether organisations and clinicians feel it works well when used alongside their current practice.

## Discussion

Prior examples of implementation planning have advocated starting early, using appropriate theory and building explicit strategies [15–17].

Implementation planning for Eczema Care Online started early and spanned all stages of its development and testing; incorporating a foundational systematic review of topical corticosteroids [26] as well as process evaluations undertaken alongside randomised controlled trials [23]. Normalisation Process Theory was used to structure and interpret data, shaping future implementation with relevant questions [11]: *is there a need for Eczema Care Online; do people want to use it; can it be easily used by different individuals; and, how will we know that it has made a difference?* Value propositions [31] were generated to distinguish Eczema Care Online from other sources of online eczema information in what was recognised to be a crowded marketplace.

With regard to research efficiencies, implementation planning reused and repurposed data generated in developing and testing Eczema Care Online, it also piggy-backed onto dissemination events (e.g. of systematic review finding – data source 2.1 and 2.2). A concern for implementation complemented all research processes, rather than being a concern to be addressed later.

These activities involved all stakeholders, including people living with eczema, parents of children who have eczema, primary care health professionals, secondary care health professionals, eczema charities and patient organisations, as well as those participating in the Eczema Care Online trials. Negotiating and incorporating different viewpoints about the potential for implementing Eczema Care Online was important, with Normalisation Process Theory providing a framework for synthesising different perspectives (Table 2).

**Table 2:** Summarises the key lessons learnt

• Start early. Implementation needs to be considered from the beginning of studies, but also consistently built up iteratively throughout the lifespan of a project, so consider resources throughout the project
• Flexibility. Likely to need to explore multiple avenues for implementation in early stages
• Secondary use of data sources. Implementation plans can be informed by multiple sources of information, some of which may already be collected for other purposes (such as process evaluation interviews)
• Consider theory but be practical too. Implementation theories such as normalization process theory can help inform plans by providing a framework. However, understanding of the landscape can help decide what is realistic with available resources, as well as following up on opportunities for implementation as they arise
• Longer term planning. Need to consider how intervention will be maintained beyond the life of the research project

Approximately two years after launching the Eczema Care Online website it has been accessed by over 50,000 people from 153 countries (as of June 2024). Links to the website have been embedded in multiple eczema charity websites, professional bodies’ websites, healthcare professional training resources, and resources used in the NHS (e.g., primary care electronic templates by Ardens [33]). The website is referenced on the NICE ‘information for the public’ webpage that supports the ‘Atopic eczema in under 12 s: diagnosis and management’ clinical guideline [34].

### Strengths and weaknesses

Implementation has been central to the project with a commitment to produce a web resource that persists beyond the research that created it. This generated new challenges for researchers who are more often concerned with delivering a clinical trial, not least an unfamiliarity with implementation theories. NPT was selected as an accessible and flexible approach, but this flexibility brings a fluidity that can make it difficult to disaggregate key aspects of implementation. Thinking of NPT on multiple levels (user, healthcare practitioner and organisational) created challenges and some of the team found the language of NPT difficult. An adapted version of the framework is in fitting with the broad approach [10] and helped it to work more specifically in the context of Eczema Care Online (Fig. 2).

It is likely that many of those stakeholders whose views informed implementation planning may have already been involved in Eczema Care Online, eczema organisations or other eczema research. Therefore, we may be missing views of important groups of people who are less engaged in eczema organisations or research.

Another possible weakness is that the team lacked marketing and commercialisation experience. A desire to ensure that Eczema Care Online remains free to access for the public is positive but creates a challenge in a financially driven health market. Whilst we have had some implementation success to date, ongoing success is contingent on resources and having funding to maintain a free at point of use intervention [35]. To date, we have been monitoring the intervention for functionality and updating content to reflect new evidence.

## Conclusion

Research projects that aim to develop an online health intervention should consider implementation early to help mitigate against the intervention falling into the ‘implementation gap’ which often happens due to lack of resources and strategy to take the idea from research to practice. Implementation requires a flexible approach, and whilst it is helpful to draw on available data and theoretical frameworks to inform strategy, understanding context specific requirements and practical constraints is necessary to make a plan that will be feasible and achievable.

## Data Availability

Authors will consider reasonable request to make relevant anonymised participant level data available.

## Abbreviations

NHS: National Health Service
NICE: National Institute of Health and Care Excellence
NIHR: National Institute of Health Research
NPT: Normalisation Process Theory

## References

[1] Ashrafzadeh S, Metlay JP, Choudhry NK, Emmons KM, Asgari MM. Using Implementation Science to Optimize the Uptake of Evidence-Based Medicine into Dermatology Practice. J Invest Dermatol. 2020;140(5):952–8. 10.1016/j.jid.2019.10.011 PMC7720814 31862108

[2] May CR, et al. Development of a theory of implementation and integration: Normalization Process Theory. Implement Sci. 2009;4(1):1–9. 19460163 10.1186/1748-5908-4-29 PMC2693517

[3] Dopson S, et al. Evidence-based medicine and the implementation gap. Health: An Interdisciplinary Journal for the Social Study of Health, Illness and Medicine. 2003;7(3):311–330.

[4] Grime P. Mind the implementation gap: the persistence of avoidable harm in the NHS. Br J Hosp Med. 2022;83(5):1–3. 10.12968/hmed.2022.0196 35653326

[5] Williams HC. Avoidable research waste in dermatology: what is the problem? Br J Dermatol. 2022;186(3):383–5. 35254683 10.1111/bjd.20754

[6] Chalmers I, Glasziou P. Avoidable waste in the production and reporting of research evidence. The Lancet. 2009;374(9683):86–9. 10.1016/S0140-6736(09)60329-9 19525005

[7] Sheridan DJ. Research: increasing value, reducing waste. The Lancet. 2014;383(9923):1123. 10.1016/S0140-6736(14)60556-0 24679621

[8] Purgar M, et al. Supporting study registration to reduce research waste. Nature Ecology & Evolution. 2024;8(8):1391–9. 38839851 10.1038/s41559-024-02433-5

[9] Ward V. Why, whose, what and how? A framework for knowledge mobilisers. Evidence and Policy. 2017;13(3):477–97.

[10] May C, Finch T. Implementing, Embedding, and Integrating Practices: An Outline of Normalization Process Theory. Sociology. 2009;43(3):535–54.

[11] Murray E, et al. Normalisation process theory: a framework for developing, evaluating and implementing complex interventions. BMC Med. 2010;8(1):63. 20961442 10.1186/1741-7015-8-63 PMC2978112

[12] Huddlestone L, et al. Application of normalisation process theory in understanding implementation processes in primary care settings in the UK: a systematic review. BMC Fam Pract. 2020;21(1):52. 32178624 10.1186/s12875-020-01107-y PMC7075013

[13] May CR, et al. Using Normalization Process Theory in feasibility studies and process evaluations of complex healthcare interventions: a systematic review. Implement Sci. 2018;13(1):80. 29879986 10.1186/s13012-018-0758-1 PMC5992634

[14] McEvoy R, et al. A qualitative systematic review of studies using the normalization process theory to research implementation processes. Implement Sci. 2014;9(1):2. 24383661 10.1186/1748-5908-9-2 PMC3905960

[15] Ross J, et al. Developing an implementation strategy for a digital health intervention: an example in routine healthcare. BMC Health Serv Res. 2018;18(1):1–13. 30340639 10.1186/s12913-018-3615-7 PMC6194634

[16] Lloyd A, et al. Patchy ‘coherence’: using normalization process theory to evaluate a multi-faceted shared decision making implementation program (MAGIC). Implement Sci. 2013;8:1–9. 24006959 10.1186/1748-5908-8-102 PMC3848565

[17] Band R, et al. Intervention planning for a digital intervention for self-management of hypertension: a theory-, evidence- and person-based approach. Implement Sci. 2017;12(1):25. 28231840 10.1186/s13012-017-0553-4 PMC5324312

[18] Greenwell K, et al. Eczema Care Online: development and qualitative optimisation of an online behavioural intervention to support self-management in young people with eczema. BMJ Open. 2022;12(4):e056867. 35443955 10.1136/bmjopen-2021-056867 PMC9021764

[19] Sivyer K, et al. Supporting families managing childhood eczema: developing and optimising eczema care online using qualitative research. Br J Gen Pract. 2022;72(719):e378–89. 35577586 10.3399/BJGP.2021.0503 PMC9119812

[20] Santer M, et al. ‘You don’t know which bits to believe’: qualitative study exploring carers’ experiences of seeking information on the internet about childhood eczema. BMJ Open. 2015;5(4):e006339. 25854963 10.1136/bmjopen-2014-006339 PMC4390694

[21] Teasdale E, Muller I, Santer M. Carers’ views of topical corticosteroid use in childhood eczema: a qualitative study of online discussion forums. Br J Dermatol. 2017;176(6):1500–7. 27753076 10.1111/bjd.15130

[22] Santer M, Muller I, Becque T, Stuart B, Hooper J, Steele M et al. Eczema Care Online behavioural interventions to support self-care for children and young people: two independent, pragmatic, randomised controlled trials BMJ 2022;379–91. 10.1136/bmj-2022-072007 PMC11778922 36740888

[23] Kate Greenwell, Katy Sivyer, Laura Howells, Mary Steele, Matthew J Ridd, Amanda Roberts, Amina Ahmed, Sandra Lawton, Sinéad M Langan, Julie Hooper, Sylvia Wilczynska, Paul Leighton, Gareth Griffiths, Tracey Sach, Paul Little, Hywel C Williams, Kim S Thomas, Lucy Yardley, Miriam Santer, Ingrid Muller, ‘Eczema shouldn’t control you; you should control eczema’: qualitative process evaluation of online behavioural interventions to support young people and parents/carers of children with eczema. Br J Dermatol. 2023;188(4):506–13. 10.1093/bjd/ljac115 36745562

[24] Greenwell K, Becque T, Sivyer K, Steele M, Denison-Day J, Howells L, Ridd MJ, Roberts A, Lawton S, Langan SM, Hooper J, Wilczynska S, Griffiths G, Sach TH, Little P, Williams HC, Thomas KS, Yardley L, Muller I, Santer M, Stuart B. Online behavioural interventions for children and young people with eczema: a quantitative evaluation. Br J Gen Pract. 2024;74(743):e379–e86. 10.3399/BJGP.2023.0411 PMC11104514 38316467

[25] Sach TH, Onoja M, Clarke H, Santer M, Muller I, Becque T, Stuart B, Hooper J, Steele M, Wilczynska S, Ridd MJ, Roberts A, Ahmed A, Yardley L, Little P, Greenwell K, Sivyer K, Nuttall J, Griffiths G, Lawton S, Langan SM, Howells L, Leighton P, Williams HC, Thomas KS. Cost-effectiveness of two online interventions supporting self-care for eczema for parents/carers and young people. Eur J Health Econ. 2024;25(7):1165–76. 10.1007/s10198-023-01649-9 PMC11377600 38194207

[26] Harvey J, et al. The long-term safety of topical corticosteroids in atopic dermatitis: A systematic review. Skin Health Dis. 2023;3(5):e268. 37799373 10.1002/ski2.268 PMC10549798

[27] National Institute for Health and Care Excellence. Atopic eczema in under 12s: diagnosis and management. 2007 [cited 2022 11th November]; Available from: https://www.nice.org.uk/guidance/cg57. 34101396

[28] SIGN. Management of atopic eczema in primary care. 2011 [cited 2022 11th November]; Available from: https://www.sign.ac.uk/our-guidelines/management-of-atopic-eczema-in-primary-care/.

[29] British Association of Dermatologists. Atopic Eczema. 2020 [cited 2021; Available from: https://www.bad.org.uk/pils/atopic-eczema/.

[30] Ritchie, J & Spencer, L 1994, ‘Qualitative data analysis for applied policy research’, in B Bryman & R Burgess (eds.), Analyzing qualitative data. London and New York: Routledge; p. 173–94.

[31] Payne A, Frow P, Eggert A. The customer value proposition: evolution, development, and application in marketing. J Acad Mark Sci. 2017;45(4):467–89.

[32] National Institute for Health and Care Excellence. Evidence standards framework (ESF) for digital health technologies. 2022 [cited 2023 15th September]; Available from: https://www.nice.org.uk/about/what-we-do/our-programmes/evidence-standards-framework-for-digital-health-technologies#how-use.

[33] https://ardens.org.uk/ - Ardens Health Informatics Ltd. 2023. [Accessed 22nd Jan 2025].

[34] National Institute for Health and Care Excellence. Atopic eczema in under 12s: diagnosis and management. 2023 [cited 2023 25th August]; Available from: https://www.nice.org.uk/guidance/cg57/ifp/chapter/More-information. 34101396

[35] Santer M, et al. How to make Eczema Care Online freely available. BMJ. 2022;379:o2973. 36740876 10.1136/bmj.o2973

